# Pretreatment prediction of pathologic complete response to neoadjuvant chemotherapy in breast cancer: Perfusion metrics of dynamic contrast enhanced MRI

**DOI:** 10.1038/s41598-018-27764-9

**Published:** 2018-06-22

**Authors:** Jeongmin Lee, Sung Hun Kim, Bong Joo Kang

**Affiliations:** Department of Radiology, Seoul St. Mary’s Hospital, College of Medicine, The Catholic University of Korea, Seoul, Republic of Korea

## Abstract

The purpose of this study was to investigate imaging parameters predicting pathologic complete response (pCR) in pretreatment dynamic contrast enhanced-magnetic resonance imaging (DCE-MRI) in breast cancer patients who were treated with neoadjuvant chemotherapy (NAC). A total of 74 patients who received NAC followed by surgery were retrospectively reviewed. All patients underwent breast MRI before NAC. Perfusion parameters including Ktrans, Kep and Ve of tumor were measured three-dimensionally. These perfusion parameters of background parenchyma of contralateral breasts were analyzed two-dimensionally. Receiver-operating characteristic (ROC) analysis and multivariable logistic regression analysis were performed to compare the ability of perfusion parameters to predict pCR. Of 74 patients, 13 achieved pCR in final pathology. The fiftieth percentile and skewness of each perfusion parameter – Ktrans, Kep, and Ve of tumor were associated with pCR. Perfusion parameters of contralateral breast parenchyma in 2D analysis also showed predictive ability for pCR. The model combining perfusion parameters of contralateral breast background parenchyma and those of the tumor had higher predictive value than each single parameter. Thus, perfusion parameters of tumor, background parenchyma of contralateral breast and their combinations in pretreatment breast MRI allow early prediction for pCR of breast cancer.

## Introduction

Locally advanced breast cancer is defined as breast cancer with large tumor size (>2 cm), regional lymph node involvement, or direct invasion of the skin or underlying chest wall^1^. In locally advanced breast cancer, neoadjuvant chemotherapy (NAC) has been proposed as the standard therapy^2,3^, because there is no difference in disease-free survival (DFS) or overall survival (OS) compared with adjuvant chemotherapy, and since the rate of breast conserving surgery can be increased^4^.

Recently, down staging of cancer and achieving pathologic complete response (pCR) after completion of NAC have emerged as challenging goals in locally advanced breast cancer, because pCR may be associated with DFS and OS^5^. Many studies have sought to identify predictive factors of pCR in breast cancer, including clinico-pathological factors and imaging parameters.

In patients who plan to receive NAC, pretreatment breast magnetic resonance imaging (MRI) is being increasingly performed, because of its high sensitivity and accuracy in detection of malignancy and clinical staging, respectively^6,7^. Morphologic features, such as tumor size and enhancement pattern, and functional parameters including perfusion parameters obtained from dynamic contrast enhanced MRI (DCE-MRI)^8–10^, and apparent diffusion cofficient (ADC) values from diffusion weighted imaging have been analyzed to predict pCR^11^.

Non-tumor factors, such as breast parenchymal enhancement (BPE) as well as tumor parameters, have been studied to investigate the correlation of tumor response after NAC. In these studies, in which either qualitative or quantitative analyses of BPE on pretreatment MRI were done, higher BPE was associated with poorer prognosis^12,13^.

Most previous studies have focused on changes in imaging parameters before and after treatment. Although it is important to know such changes, identifying the factor that can predict pCR with pretreatment images will truly allow early prediction of pCR. Early and accurate prediction of tumor response would enable proper choice of treatment regimen and proper operative planning by the surgeon.

We investigated imaging parameters for early prediction of pCR with pretreatment DCE-MRI, focusing on tumor perfusion parameters and background parenchymal enhancement of contralateral breast parenchyma.

## Results

### Patients

A total of 74 breast cancer patients who underwent pretreatment DCE-MRI with surgery after NAC were included. After completion of NAC, 22 patients underwent breast conservative surgery (BCS) and 52 patients underwent mastectomy. Immunohistochemical staining of final surgical specimens revealed luminal type in 47 cases, HER-enriched type in 14 cases, and triple-negative type in 13 cases.

Thirteen (17.5%) of these 74 patients achieved pCR based on specimens obtained from surgery after NAC. Of these 13 patients, seven were confirmed to be luminal type while six as HER2+ type. There was no triple negative type. Cancer staging was classified according to AJCC 7th. Stage 0 was the most common (nine of 13 patients). The remaining four patients had lymphovascular invasion in final pathology. They were classified as stage I (n = 1), II (n = 2), and III (n = 1) (Table 1).

**Table 1:** Patient demographics.

	Total (n = 74)	
	CR (n = 13)	Non-CR (n = 61)
Age		
Median (range)	49.0 (37.0–66.0)	45.0 (25.0–67.0)
Subtype		
luminal A, B	7	40
Her2^+^	6	8
Triple negative	—	13
Pathological TNM		
0	9	—
I	1	21
II	2	24
III	1	16
Surgery type		
Breast conservative surgery (BCS)	5	17
Mastectomy	8	44

### Predictive ability of perfusion parameters

#### Tumor and background parenchyma of contralateral breast

Each perfusion parameter of DCE-MRI in tumor and background parenchyma of contralateral breast (BP_CL_) did not show high predictive ability for pCR based on receiver operating characteristic (ROC) curve analysis (AUC < 0.7, 0.449 to 0.683) (Table 2). However, mean Ktrans value of BP_CL_ (AUC = 0.683) had relatively better predictive ability than other single perfusion parameters.

**Table 2:** Diagnostic performance of perfusion parameters from DCE-MRI to predict pathologic complete response.

	Cut-off value*	non-CR (n = 61)	CR (n = 13)	Sensitivity (99.8% CI**)	Specificity (99.8% CI**)	AUC (99.8% CI**)
**2D analysis of BP**_**CL**_						
Ktrans	≥0.03	16	8	0.615 (0.190–0.940)	0.738 (0.560–0.915)	0.683 (0.425–0.941)
Kep	≥0.21	12	6	0.462 (0.025–0.795)	0.803 (0.643–0.964)	0.627 (0.331–0.924)
Ve	≥0.20	11	6	0.462 (0.025–0.795)	0.820 (0.664–0.975)	0.629 (0.332–0.925)
**3D histogram analysis of tumor**						
Ktrans						
25th perc	≥0.11	29	9	0.692 (0.288–1.000)	0.525 (0.323–0.726)	0.610 (0.335–0.885)
50th perc	≥0.22	24	9	0.692 (0.288–1.000)	0.607 (0.409–0.804)	0.624 (0.305–0.943)
75th perc	≥0.34	19	9	0.692 (0.288–1.000)	0.689 (0.501–0.876)	0.612 (0.285–0.938)
mean	≥0.24	23	9	0.692 (0.288–1.000)	0.623 (0.427–0.819)	0.605 (0.287–0.924)
kurtosis	≤−0.92	28	4	0.308 (0.000–0.616)	0.541 (0.340–0.742)	0.502 (0.235–0.769)
skewness	≤0.52	38	12	0.923 (0.690–1.000)	0.377 (0.181–0.573)	0.647 (0.388–0.905)
Kep						
25th perc	≥0.14	50	13	1.000 (1.000–1.000)	0.180 (0.025–0.336)	0.546 (0.267–0.825)
50th perc	≥0.51	22	8	0.615 (0.190–0.940)	0.639 (0.445–0.833)	0.575 (0.257–0.893)
75th perc	≥0.94	9	5	0.385 (0.000–0.710)	0.852 (0.709–0.996)	0.571 (0.241–0.902)
mean	≥0.55	24	8	0.615 (0.190–0.940)	0.607 (0.409–0.804)	0.574 (0.250–0.897)
kurtosis	≥4.53	2	2	0.154 (0.000–0.395)	0.967 (0.895–1.000)	0.508 (0.212–0.804)
skewness	<0.82	41	10	0.769 (0.400–1.000)	0.328 (0.138–0.518)	0.535 (0.267–0.802)
Ve						
25th perc	≤0.43	31	9	0.692 (0.288–1.000)	0.492 (0.290–0.694)	0.472 (0.243–0.702)
50th perc	≤0.52	28	9	0.692 (0.288–1.000)	0.541 (0.340–0.742)	0.487 (0.236–0.738)
75th perc	≤0.61	30	8	0.615 (0.190–0.940)	0.508 (0.306–0.710)	0.484 (0.223–0.745)
mean	≤0.53	29	9	0.692 (0.288–1.000)	0.525 (0.323–0.726)	0.494 (0.244–0.745)
kurtosis	≤0.28	40	6	0.462 (0.025–0.795)	0.344 (0.152–0.536)	0.449 (0.183–0.715)
skewness	≤−0.10	23	9	0.692 (0.288–1.000)	0.623 (0.427–0.819)	0.641 (0.375–0.907)

*Optimal cut off point was obtained from Youden index on ROC curve perc, percentile.

**Confidence interval use a Bonferroni corrected (1–0.05/30) confidence level.

#### Combination of parameters from tumor and background parenchyma of contralateral breast

Parameters with relatively high AUC values in 3D histogram analysis were extracted, including 50th percentile values and skewness of Ktrans, Kep, and Ve values. These parameters were combined with perfusion parameters of BP_CL_.

The model combining perfusion parameters of BP_CL_ and those of tumor showed higher predictive value than each single parameter. The combination of Ve of BP_CL_ with 50th percentile and the skewness of Ve in tumor had the highest predictive power for pCR (AUC = 0.807, *p* = 0.002). The AUC value of a combination of Ktrans of BP_CL_ and skewness of Ktrans in tumor was 0.760 (*p* = 0.003), and that of a combination of all three parameters – Ktrans of BP_CL_, 50th percentile Ktrans in tumor, and skewness of Ktrans in tumor – was 0.757 (*p* = 0.004). Those showed higher predictive ability for pCR than each single parameter. Other combinations of parameters also showed high predictive power with AUC > 0.7, although they were not statistically higher than each single parameters. These included a combination of Ktrans of BP_CL_ and 50th percentile Ktrans of tumor (AUC = 0.731, *p* = 0.092), and a combination of Ve of BP_CL_ and skewness of Ve in (AUC = 0.718, *p* = 0.061) (Table 3).

**Table 3:** AUC valus and p value of the model combining perfusion parameters of contralateral breast background parenchyma and those of the tumor.

	Combination of parameters		AUC (99.8% CI**)	Bonferroni-corrected *p* value
	2D analysis	3D histogram analysis		
Ktrans	Background parenchyma of contralateral breast	50percentile	0.731 (0.485–0.978)	0.092
		skewness	0.760 (0.549–0.972)	0.003
		50percentile + skewness	0.757 (0.543–0.970)	0.004
Kep	Background parenchyma of contralateral breast	50percentile	0.626 (0.329–0.922)	>0.999
		skewness	0.633 (0.339–0.927)	>0.999
		50percentile + skewness	0.631 (0.336–0.925)	>0.999
Ve	Background parenchyma of contralateral beast	50percentile	0.628 (0.328–0.928)	>0.999
		skewness	0.718 (0.495–0.940)	0.061
		50percentile + skewness	0.807 (0.563–1.000)	0.002

**Confidence interval use a Bonferroni corrected (1–0.05/30) confidence level.

Univariate and multivariable logistic regression analyses were performed to investigate the correlation between MRI perfusion parameters and pCR. All individual perfusion parameters were cateogrized as 0 or 1, based on optimal cutoff values obtained through ROC analysis. Combination models were scored by the sum of categorized single perfusion parameters (Table 4). In multivariable logistic regression analysis, odds ratio (OR) of each single perfusion parameter was not associated with pCR, except Ktrans of BP_CL_ (OR = 0.01, 95% CI: < 0.001–0.55, *p* = 0.023) (Table 5). However, combination models showed much higher association with pCR than single parameters, with higher OR and statistical significance. The model with a combination of Ktrans of BP_CL_ and skewness of Ktrans in tumor showed the highest OR (5.98, 95% CI: 1.89–18.97, *p* = 0.002). The model with a combination of all three parameters of Ve also showed a high OR (5.26, 95% CI: 1.89–14.64, *p* = 0.002) (Table 6).

**Table 4:** Scoring of model with a combination of perfusion parameters.

	Combining model	Scoring			
		0	1	2	3
Ktrans	BP_CL_ & 50th percentile	<0.03 & <0.22	≥0.03^†^ or ≥0.22^†^	≥0.03^†^ & ≥0.22^†^	—
	BP_CL_ & skewness	<0.03 & >0.52	≥0.03^†^ or ≤0.52^†^	≥0.03^†^ & ≤0.52^†^	—
	BP_CL_ & 50 percentile & skewness	<0.03 & <0.22 & >0.52	One of ≥0.03^†^ or ≥0.22^†^ or ≤0.52^†^	Two of ≥0.03^†^ or ≥0.22^†^ or ≤0.52^†^	≥0.03^†^ & ≥0.22^†^ & ≤0.52^†^
Kep	B P_CL_ & 50th percentile	<0.21 & <0.51	≥0.21^†^ or ≥0.51^†^	≥0.21^†^ & ≥0.51^†^	—
	BP_CL_ & skewness	<0.21 & ≥0.82	≥0.21^†^ or <0.82^†^	≥0.21^†^ & <0.82^†^	—
	BP_CL_ & 50 percentile & skewness	<0.21 & <0.51 & ≥0.82	One of ≥0.21^† †^ or ≥0.51^†^ or <0.82^†^	Two of ≥0.21^†^^†^ or ≥0.51^†^ or <0.82^†^	≥0.21^††^ & ≥0.51^†^ &<0.82^†^
Ve	BP_CL_ & 50th percentile	<0.20 & >0.52	≥0.20^†^ or ≤0.52^†^	≥0.20^†^ & ≤0.52^†^	—
	BP_CL_ & skewness	<0.20 & >−0.10	≥0.20^†^ or ≤−0.10^†^	≥0.20^†^ & ≤−0.10^†^	—
	BP_CL_ & 50th percentile & skewness	<0.20 & >0.52 & >−0.10	One of ≥0.20^†^ or ≤0.52^†^ or ≤−0.10^†^	Two of ≥0.20^†^ or ≤0.52^†^ or ≤−0.10^†^	≥0.20^†^ & ≤0.52^†^ & ≤−0.10^†^

^†^The optimal cut off point was obtained from Youden index on ROC curve.

**Table 5:** Uni- and multivariable logistic regression of single perfusion parameters as categorical variables.

	crude odds ratio(95% CI)	*p* value	adjust odds ratio(95% CI)	*p* value
***2D analysis of perfusion parameters of BP***_***CL***_				
BP_CL_ _Ktrans, ≥0.03	4.50 (1.28–15.78)	0.019	0.01 (<0.001–0.55)	0.023
BP_CL_ _Kep, ≥0.21	3.50 (0.99–12.34)	0.051	0.21 (0.02–2.15)	0.188
BP_CL_ _Ve, ≥0.20	3.90 (1.09–13.89)	0.036	1.11 (0.07–18.40)	0.945
***3D analysis of perfusion parameters of tumor***				
*Ktrans*				
50perc, ≥0.22	3.47 (0.96–12.54)	0.058	0.68 (0.03–13.51)	0.797
skewness, ≤0.52	7.26 (0.89–59.58)	0.065	0.00 (<0.001–0.51)	0.028
*Kep*				
50perc, ≥0.51	2.84 (0.83–9.74)	0.098	0.10 (0.01–1.14)	0.064
skewness < 0.82	1.63 (0.40–6.57)	0.495	1.97 (0.14–28.50)	0.619
*Ve*				
50perc, ≤0.52	2.65 (0.74–9.55)	0.136	0.01 (<0.001–0.27)	0.008
skewness, ≤−0.10	3.72 (1.03–13.46)	0.046	0.14 (0.01–1.28)	0.081

**Table 6:** Univariable logistic regression of combining model of perfusion parameters.

	Adjusted Odds ratio (95% CI)	*p*-value
***Combining model of Ktrans***		
BP_CL_ and 50 percentile	3.13 (1.33–7.35)	0.009
BP_CL_ and skewness	5.98 (1.89–18.97)	0.002
BP_CL_ and 50 percentile and skewness	2.91 (1.40–6.03)	0.004
***Combining model of Kep***		
BP_CL_ and 50 percentile	2.56 (1.13–5.84)	0.025
BP_CL_ and skewness	2.66 (0.95–7.48)	0.064
BP_CL_ and 50 percentile and skewness	2.34 (1.10–4.98)	0.027
***Combining model of Ve***		
BP_CL_ and 50 percentile	4.82 (1.46–15.90)	0.010
BP_CL_ and skewness	2.85 (1.25–6.48)	0.013
BP_CL_ and 50 percentile and skewness	5.26 (1.89–14.64)	0.002

## Discussion

In locally advanced breast cancer, NAC has been suggested as a means to improve prognosis. Patients who acquired pCR after NAC can expect better DFS or OS compared to patients with non-complete response^14^. Many studies have investigated imaging parameters of MRI with the aim to predict pCR in breast cancer patients who undergo NAC. Using conventional breast MRI, initial tumor size, tumor size reduction, and ADC value have been suggested as predictive factors of pCR in pre- and post-treatment MRI, after four cycles of NAC^15^.

However, conventional breast MRI cannot reflect neoangiogenesis of the tumor, which is one of the most important prognostic factors of breast cancer. Tumor neoangiogenesis reflects dissemination of malignant cells as well as the level of contrast enhancement. Imaging parameters capable of revealing vascular kinetics of tumors are important to monitor effects of chemotherapy and predict tumor prognosis^16,17^. DCE-MRI allows quantitative measurements of kinetic parameters related to perfusion and permeability of tumor. Studies using DCE-MRI have investigated imaging parameters with the goal to predict tumor response and prognosis. Martin D. Pickles *et al*. have suggested that patients with high perfusion and vascular permeability in pretreatment DCE-MRI are significantly less likely to have long-term DFS and OS as a result of neovascularization^18^. In a subsequent study, the authors also suggested that quantification of perfusion parameters could provide information to distinguish responders from non-responders before finishing the treatment, by demonstrating that early treatment change was possible for non-responders^19^. However, these studies focused on changes of MRI imaging parameters before and after NAC as a means to predict pCR. Post-treatment MRI must be performed for prediction, which is expensive. In addition, the predictive factors presented in these studies could not be considered as true “predictive” factors because they were determined as predictors after at least one cycle of chemotherapy.

Our data demonstrate that multiple perfusion parameters obtained from pretreatment DCE-MRI can predict pCR, especially when single parameters are combined. Perfusion parameter of BP_CL_ was explored using 2D analysis and tumor perfusion parameters were assessed by 3D analysis. Perfusion parameter Ktrans of BP_CL_ displayed the highest AUC (0.683) in 2D analysis, while the skewness of Ktrans displayed the highest AUC value (0.647) in 3D analysis of tumor. The AUC value of these combined perfusion parameters had a higher predictive ablity for pCR than each single parameter by ROC analysis. Multivariable logistic regression analysis of perfusion parameters that were combined after categorization revealed much higher association with pCR than each single perfusion parameter showing increased OR and statistical significance.

The present results indicate that perfusion parameters of BP_CL_ could be a predictive factor for pCR. The relationship between BPE and tumor response has already been studied with a focus on qualitative or quantitative BPE reduction before and after NAC^20,21^. Higher BP_CL_ is associated with worse prognosis, such as early tumor recurrence^12,22^. However, since these studies involved qualitative categorization of BPE rather than quantitative measurement, there might be a limit to use of BPE as an objective index for predicting tumor response. We used perfusion parameters derived from pretreatment DCE-MRI to investigate the relationship between tumor response and BP_CL_. Each single perfusion parameter of BP_CL_ showed fair ability to predict pCR, with a higher predictive ability when tumor perfusion parameters are combined.

This study has some limitations. First, it was a retrospective study with a small number of patients. The number of patients with pCR was much smaller than that of patients with non-pCR. Therefore, our results might not be generalizable. Second, patients received pretreatment MRI regardless of their menstrual cycle, because our study was a retrospective study. This would not be a limitation in postmenopausal patients. However it could affect perfusion parameters of BPE in contralateral breast parenchyma in premenopausal patients. Third, we excluded bilateral breast cancer, because it would be difficult to measure perfusion parameters of the contralateral breast parenchyma.

We investigated the correlation between pretreatment MRI parameters and postoperative final pathology without long-term follow-up after surgery. Thus, DFS and OS, including early or late tumor recurrence which could be correlated with pCR after NAC, were not investigated. Additional study will be needed to investigate imaging parameters in pretreatment MRI to predict DFS and OS after NAC.

In conclusion, early predictive factors of pCR in breast cancer patients using pretreatment MRI only, were explored. Each single perfusion parameters of tumor and BP_CL_ showed fair predictive power for pCR of breast cancer. However, the combination of both perfusion parameters of tumor and BP_CL_ showed higher predictive power for pCR of breast cancer.

## Material and Methods

### Patients

**Figure 1:**
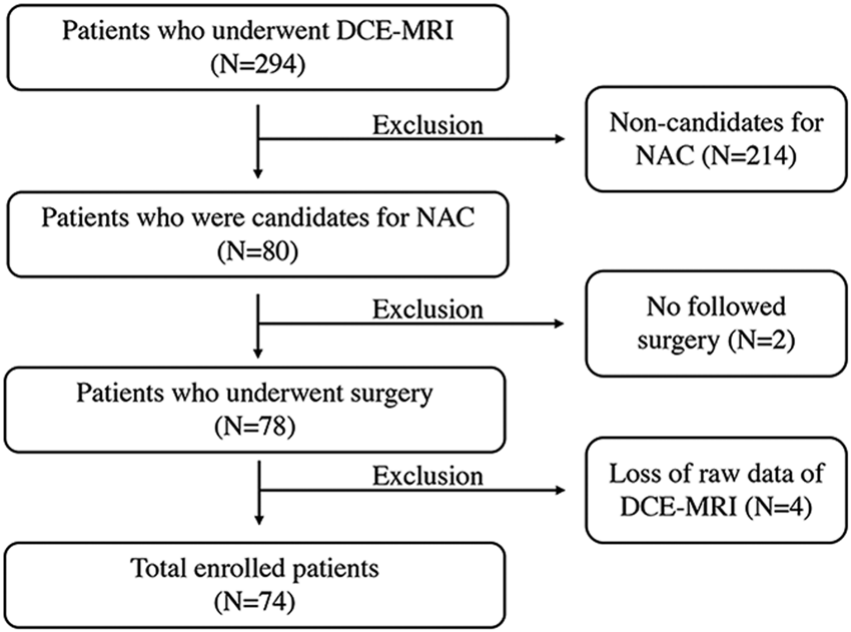
Patient inclusion diagram.

This study was approved by the Institutional Review Board of Seoul St. Mary’s Hospital. It was performed in accordanace with procedures complied with HIPAA guidelines. The requirement for informed consent was waived due to its retrospective nature. A total of 294 breast cancer patients who underwent DCE-MRI between February 2014 and May 2016 were included. Of them, 214 patients who were not candidates of NAC before surgery were excluded. Two patients were also excluded from the remaining 80 patients because they had not received breast surgery. Of the remaining 78 patients, DCE-MRI raw data for postprocessing analysis were lost for four patients. Finally, 74 patients were enrolled (Fig. 1).

### NAC

All patients underwent pretreatment MRI before the first cycle of NAC. All 74 patients received anthracycline-based chemotherapy. Twenty-nine patients were treated with a combination of anthracycline and cyclophosphamide, of which seven underwent additional Herceptin use. Forty-five patients were treated with the combination of anthracycline and taxane, of which seven underwent additional Herceptin treatment. After completion of NAC, all patients underwent surgery including breast conserving surgery or total mastectomy.

### Pathology

Pathologic complete response (pCR) was defined as the absence of any invasive component, or residual ductal carcinoma *in situ* (DCIS) component in the specimen obtained from surgery^23,24^. A non-pCR was defined as the presence of microscopic invasive tumor in the final pathology^23^. All pathological information including immunohistochemical staining was obtained from surgically obtained specimens. However, immunohistochemical staining results were obtained from biopsy samples for pCR patients who did not even have a residual DCIS component in surgical specimen.

### MRI

MRI examinations were performed for patients in prone position using a Magnetom Verio 3 T system (Siemens Healthcare, Erlangen, Germany) and a dedicated eight-channel phase-array coil. Images were obtained using the following sequences: (1) axial turbo spin-echo T2-weighted imaging (T2WI) with TR/TE of 4530/93 msec, flip angle of 80°, field of view (FOV) 320 × 320 mm^2^, matrix size of 576 × 403, slice thickness of 4 mm, and acquisition time of 2 min 28 sec; (2) pre-contrast T1-weighted three-dimensional (3D) volumetric interpolated breath-hold examinations (3D VIBE) with TR/TE of 2.7/0.8 msec, FOV of 320 × 320 mm^2^, matrix size of 256 × 192, slice thickness of 2 mm with various flip angles (2°, 6°, 9°, 12°, 15°), and acquisition time of 2 min 15 sec to determine tissue T1 relaxation time prior to the arrival of contrast agent; (3) dynamic contrast-enhanced axial T1-weighted imaging (T1WI) with fat suppression with TR/TE of 2.5/0.8 msec, flip angle of 10°, slice thickness of 2.0 mm, and acquisition time of 5 min 30 sec (temporal resolution 6 sec) following an intravenous bolus injection of 0.1 mmol/kg gadobutol (Gadovist, Schering, Berlin, Germany) followed by a 20 ml saline flush; (4) delayed axialT1-weighted 3D VIBE with TR/TE of 4.4/1.7 msec, flip angle of 10°, slice thickness of 1.2 mm, FOV of 340 mm, and matrix size of 448 × 358 to evaluate the overall extent of tumor.

### Imaging analysis

To evaluate perfusion parameters from DCE-MRI, a standard Tofts model was used. Parameters such as volume transfer rate (Ktrans [min^−1^]), volume of extravascular extracellular space (EES) per unit volume of tissue (Ve), and flux rate constant between EES and plasma (Kep, [min^−1^])^25^ were calculated using postprocessing software (Olea Sphere, version 3.0, Olea Medical, La Ciotat, France). In Olea Sphere, it was chosen to derive the arterial input function (AIF) from perfusion weighted image pixels selected in ascending aorta, and the AIF was calculated automatically. Analysis of perfusion parameter was performed for the tumor itself, and the background parenchyma of the breast on the contralateral side without tumor involvement.

#### Tumor

**Figure 2:**
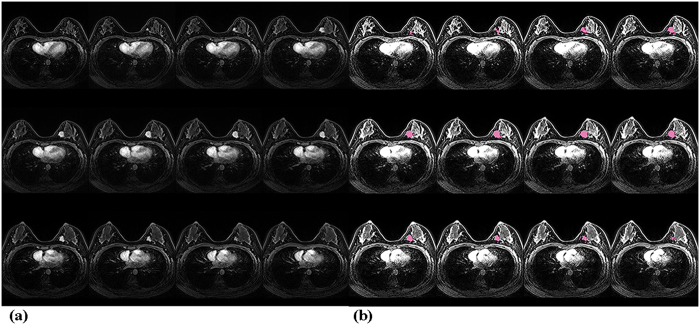
(**a**) Fat-saturated T1-weighted images with gadolinium enhancement in a patient with breast cancer at the mid-inner portion of the left breast. (**b**) Applying magic wand tool in post processing program (Olea Sphere, version 3.0) to extract and analyze the enhancing portion of the tumor in the same patient.

Perfusion parameters of the tumor were obtained by 3D histogram analysis. In the analysis, after the whole outline of tumor was drawn manually, magic wand, a semi-automated region-growing segmentation tool was used to find and analyze the enhancing portion of the tumor (Fig. 2a,b). The 25th, 50th, 75th percentile, mean value, and skewness and kurtosis of each Ktrans, Kep, and Ve were then obtained.

#### Background parenchyma of contralateral breast

**Figure 3:**
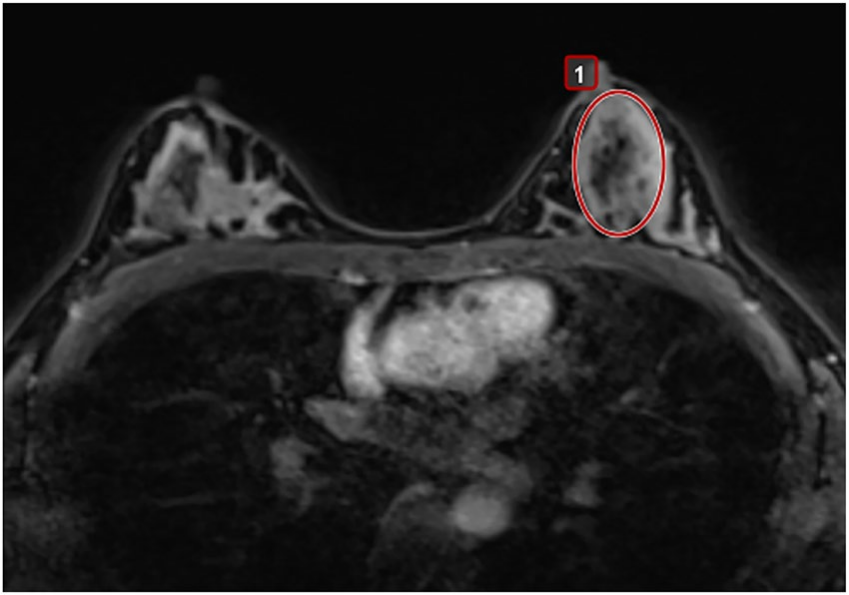
Applying a eliptical region of interest (ROI) in post processing program (Olea Sphere, version 3.0) to obtain perfusion parameter of background parenchyma of the contralateral breast.

Perfusion parameters of BP_CL_ were also obtained by two-dimensional (2D) analysis. Mean values of Ktrans, Kep, and Ve were derived. These parameters were measured by drawing a eliptical region of interest (ROI) at the level showing the largest fibroglandular tissue without enhancing lesions, except for the skin and fat layer (Fig. 3).

### Statistical analyses

To test the predictive ability of each single perfusion parameter for pCR based on DCE-MRI, ROC curve analysis was performed. Optimal cut-off values, sensitivities, and specificities of each parameter were obtained from the Youden index on the ROC curve.

A multivariate logistic regression model combining perfusion parameters of BP_CL_ and those of the tumor were fitted and their performance was evaluated by area under the curve (AUC). Univariate and multivariate logistic regression models were used to calculate OR of converting perfusion parameters with optimal cutoff. Additionally, univariate logistic regression was perfomed to gauge the effect of combined perfusion parameters (Table 4). A Bonferroni correction was applied for multiple comparisons, with a correction factor derived from the number of perfusion parameters. For comparison of total 30 parameters – 21 parameters of each single perfusion parameters (Table 2) and 9 combining perfusion parameters (Table 3), critical *p* value was calculated as 0.0017 from 0.05/30.

All statistical analyses were performed using SAS ver. 8.4 software (SAS Institute Inc., Cary, NC, USA). A *p* value < 0.05 was considered statistically significant.
